# The Application of Ultrasound and Enzymes Could Be Promising Tools for Recovering Polyphenols during the Aging on Lees Process in Red Winemaking

**DOI:** 10.3390/foods11010019

**Published:** 2021-12-22

**Authors:** Andrea Osete-Alcaraz, Ana Belén Bautista-Ortín, Paula Pérez-Porras, Encarna Gómez-Plaza

**Affiliations:** Department of Food Science and Technology, Faculty of Veterinary Science, Campus de Espinardo, University of Murcia, 30100 Murcia, Spain; andrea.osete@um.es (A.O.-A.); anabel@um.es (A.B.B.-O.); paula.perez2@um.es (P.P.-P.)

**Keywords:** lees, ultrasound, β-glucanase, pectinase, anthocyanins, tannins

## Abstract

The final concentration of phenolic compounds in wines is usually lower than what might be expected, given their concentration in grapes. This is in part due to the interactions between cell walls from grapes and yeast with phenolics during red winemaking. Most of these aggregates are insoluble and end up precipitating, forming part of the lees. The objective of this study is to determine the capacity of ultrasounds and/or enzymes treatments (β-glucanase and a pectolytic enzyme) to release the anthocyanins and tannins adsorbed in the lees. The ultrasound (US) applied for 120 min slightly favored the extraction of anthocyanins and doubled tannin extraction. Shorter sonication times did not show any positive effect. The combination of β-glucanase and pectolytic enzyme was always more effective in the liberation of anthocyanins (both no-acylated and acylated anthocyanins) and tannins than the enzymes acting separately. The combination of US (120 min), β-glucanase and pectolytic enzyme showed an additive effect, increasing the extraction of phenolic compounds with respect to the individual treatments and also releasing a large quantity of low molecular weight polysaccharides, compounds of enological importance. These results of this study could be of enological interest, facilitating and accelerating the aging on lees process, through the liberation of polysaccharides and the recovery of the phenolic compounds lost during vinification.

## 1. Introduction

Wine lees are formed by the combination of dead yeasts, their metabolites and phenolic compounds, together with tartaric salts, bacteria and debris from plant cells [1].

The main component of the *Saccharomyces cerevisiae* cell wall is β-(1 → 3) glucan, which form its main chain, and presenting β-(1 → 6) glucan lateral ramifications and chitin [2,3]. These compounds create the tridimensional structure that support the glucomannan–protein complex [4]. During the autolysis of the yeasts, their cell walls are gradually degraded due to the breakage of the glucan and chitin chain. This is performed by the action of mannosidases and glucanases, enzymes which belong to the dead yeasts themselves [5].

Aging on lees in red wines has become a popular technique nowadays. It consists of keeping the wine in barrels or tanks to which the lees recovered after alcoholic and/or malolactic fermentations has been added. The wine is regularly stirred, with greater or lesser frequency, to promote the transference of compounds from the lees to the wine. Due to the autolysis, the yeast cell walls become less rigid, and their polysaccharides are released [6]; these compounds may improve the color, protein and tartaric stability of wines [7,8] and reduce their astringency [9]. Together with the polysaccharides, hydrolyzed proteins are also liberated, increasing the content of nitrogenous compounds in wine [10] as well as lipids that contribute the aromatic fraction of the wine, since they can be precursors to the formation of esters and aldehydes [11]. Therefore, the aim of this technique is to obtain more complex quality wines with improved organoleptic characteristics [12].

Less attention has been paid to the fact that lees can also be a source of phenolic compounds. These phenolic compounds are retained both by yeast cell walls and by grape skin and pulp cell walls. In the case of yeasts, and as previously mentioned, the *Saccharomyces cerevisiae* cell wall is made up of mannoproteins linked to oligosaccharides, which remain exposed outside the cell. The different polarities and the hydrophilic or hydrophobic nature of these wall polymers define the ability of yeasts to retain or adsorb different wine molecules as phenolic compounds or volatile compounds [13,14,15]. In addition, the porosity of the wall also influences the adsorption, since the greater contact surface provided by the interstitial spaces provides a greater amount of attachment points for the phenolic compounds [16]. Furthermore, Mekoue-Nguela et al. [17,18] reported that not only the yeast cell walls had a role in the adsorption of polyphenols such as tannins, but that they also diffuse freely through the walls of dead cells to interact with their plasma membrane and cytoplasmic components.

On the other hand, at the beginning of the vinification, there is an important amount of suspended vegetal material, and their cell walls also present high affinity for phenolic compounds. The binding of phenolic compounds to the cell walls of this suspended plant material leads to high molecular weight complexes that are insoluble and end up precipitating in later stages of the vinification process and then become part of the lees [19]. Moreover, wine polyphenols may also form complexes with other macromolecules that appeared in wine lees, including proteins [20,21]. Therefore, the precipitate lees present a considerable amount of adsorbed phenolic compounds, and hence, aging on lees, in addition to the extraction of compounds of interest such as mannoproteins and aroma precursor compounds, could also increase the phenolic composition of red wines if the phenolic compounds bound to them could be desorbed. To this end, no information can be found as to whether the biochemical and physical treatments that are normally used to degrade the components of red wine lees may also help to liberate these phenolic compounds.

Among these tools, β-glucanase is commonly used. It is a hydrolytic enzyme that degrades the β-glucans, one of the main components of the cell wall of the Saccharomyces cerevisiae yeast. Enzymatic preparations with β-glucanase have been used in order to accelerate the release of polysaccharides and mannoproteins during the aging on lees [22]. Taking into account that the main drawback of aging on lees is that it prolongs wine processing times (several months are necessary to obtain perceptible effects), β-glucanase treatments allow to reduce the elaboration costs by shortening aging on lees time [23]. The possible role of this enzyme in the release of the phenolic compounds bound to them has not been reported.

On the other hand, and since plant cell walls also are present in the lees, the possible role of pectinase enzymes, whose main enzyme activities are methylesterase, polygalacturonase and pectin-lyase, on the desorption of phenolic compounds needs to be addressed. These enzymes facilitate the degradation of the pectin fraction of the grape cell wall that is part of the lees [24], and this degradation may release low molecular weight polysaccharides to the wine and help the liberation of polyphenols [25,26].

Another technique that could help the extraction of the bound phenolic compounds during aging on lees is ultrasound (US). This non-thermal technique is being used in wine industry for its ability to accelerate the extraction of compounds from inside the grape cells, shortening maceration time in the wineries. This treatment is based on the use of mechanical waves of 16–100 kHz to produce physical-chemical changes in the matrix where they are applied [27]. The cavitation phenomenon, that is, the formation and collapse of bubbles produced by the movements of compression and expansion of the molecules of a liquid which are caused by the ultrasonic waves, produces these changes. Moreover, the implosion of the bubbles near the cell walls causes the disruption of the cells which improves the liberation of the compounds contained inside the cells [28]. The high temperatures and pressures that are locally reached due to cavitation could help the release of polyphenols adsorbed in the cell walls. Moreover, ultrasound could desorb the polyphenols that are bound to cell walls forming multilayers, as hypothesized by Beaver et al. [29], and this could facilitate the access of the enzymes that degrade the cell wall.

As stated before, US has been previously used during the early stages of red wine production in order to accelerate the extraction of phenolic compounds from within the cell, reducing maceration times and improving the phenolic composition of red wines [28,30,31]. This technology has also been used to accelerate aging on lees [32,33,34]. These authors especially focused on the acceleration of polysaccharide extraction during aging on lees when USs were used. They achieved a reduction in lees aging duration of 2–3 weeks. However, we cannot discard that ultrasound treatment may lead to the formation of radical compounds that could accelerate some degradation reactions in wine [35].

As regards the recovery of phenolic compounds, enzymes and ultrasound treatments have been used before for the recovery of anthocyanins or glycoproteins from red wine lees, considering the lees as a winemaking byproduct. However, in these studies, the solvents used to perform the extraction were mixtures of ethanol or methanol in water at high concentrations, which have a more efficient extraction capacity than the wine (a hydroalcoholic solution with 12–15% of ethanol), and thus, far from the conditions of real winemaking [36,37].

Although the use of enzymatic and US treatments for accelerating the autolysis of yeasts, and therefore the extraction of polysaccharides and mannoproteins, have been previously studied, no attention has been paid to their possible contribution to the release of phenolic compounds, such as anthocyanins and tannins, adsorbed in the precipitated lees. Therefore, with this objective, ultrasound, pectinases and β-glucanase were applied alone or in combination to a model solution containing suspended freeze-dried lees obtained from a red wine vinification, and the release of anthocyanins, tannins and polysaccharides was investigated.

## 2. Materials and Methods

### 2.1. The Preparation of the Lees

Wine lees were recovered from a traditional red wine produced in the experimental cellar of the University of Murcia. At the end of alcoholic fermentation, the lees were recovered, washed twice with distilled water and centrifuged for 5 min at 3500 rpm. Then, they were stored at −18 °C until they were lyophilized and grounded to a fine powder (<0.6 mm).

### 2.2. Enzyme and Ultrasound Treatments

The desorption tests were carried out in 12 mL glass tubes with a screw cap. A total of 0.5 g of lyophilized lees and 8 mL of a hydroalcoholic solution (12% ethanol at pH 3.6 adjusted with triphloroacetic acid) were added to each tube. Then, the different treatments were applied.

Control: The control sample preparation was carried out by a simulated bâtonnage as was proposed by Liu et al. [38]

Enzymatic treatments. The pectolytic enzyme (PEC) was added at a final concentration of 0.03 mL/L (EnozymLux, Agrovin S.A., Alcázar de San Juan, Spain). The main enzymatic activities of this preparation (as provided by the supplier) are polygalacturonase (4500 U/g), pectin methyl esterase (1000 U/g) and pectin-lyase (PL) (130 U/g). The glucanase enzyme (GLUC) was added at a final concentration of 0.05 g/L (EnozymGLUCAN, Agrovin S.A., Alcázar de San Juan, Spain). The main enzymatic activity of this preparation is β-1,3-1,6 glucanase (10,000 U/g). Enzyme preparations were added individually (PEC and GLUC) and in combination (PEC + GLUC). After its addition, the tube was stirred in an orbital shaker at 300 rpm for 24 h.

Ultrasound treatment. The application of ultrasound (US) was carried out using the equipment conditions reported by Kulkarni et al. [33], using those when these authors found the highest polysaccharide release from lees as treatment times.

Three treatment times were tested: 30, 60 and 120 min (US30′, US60′ and US120′). The tubes were placed in an ultrasonic bath (Branson 8800 Ultrasonic cleaner, Branson Ultrasonic Corp., Danbury, CT, USA, 25 L), which operates at a frequency of 40 kHz, a power of 280 W and at a controlled temperature of 18 ± 1 °C.

Enzyme and Ultrasound treatment combinations. The treatment time that showed the highest extraction of anthocyanins and tannins (as will be later discussed) from the lees was 120 min, and, therefore, it was chosen as the treatment time in the combinations of enzymatic and ultrasound treatments. Two different combinations were assayed, with the US being applied before or after the addition of the enzymes.

All treatments and combinations were performed in triplicate. After 24 h, the tubes were centrifuged for 5 min at 4000 rpm, the supernatant was recovered and filtered with 0.45 μm nylon filters. In total, 2.5 mL of the filtered supernatant was used for the analysis of tannins by liquid chromatography (HPLC) and size-exclusion chromatography (SEC), and 2 mL for the analysis of soluble polysaccharides by SEC.

### 2.3. Tannin Analysis by Liquid Chromatography Using the Phloroglucinolysis Method

The composition and quantification of the tannins released from the lees were analyzed by the phloroglucinolysis reaction, according to the method described by Del Rio and Kennedy [39], with some modifications derived from the type of sample, which are described below.

In total, 2.5 mL of filtered supernatant (0.45 µm nylon filter) was concentrated in a Centrivap vacuum concentrator (Labconco, Kansas City, MO, USA) after which it was dissolved in 250 µL of methanol. Then, 50 μL of the methanol extract was mixed with 50 μL of the phloroglucinolysis reagent prepared as described by Osete-Alcaraz et al. [28]. The mixture was heated in a 50 °C water bath for 20 min. To stop the reaction, 100 µL of an aqueous sodium acetate solution (0.2 M) was added, after which they were centrifuged at 10,000 rpm for 5 min. The injection volume was 10 µL. The conditions of the HPLC analysis were described by Osete-Alcaraz et al. [28].

### 2.4. Tannin Analysis by Size Exclusion Chromatography (SEC)

Size exclusion chromatography (SEC) was also used to analyze tannins released from lees. This method was described by Kennedy and Taylor [40], with some adaptations described by Castro-López et al. [41].

### 2.5. Anthocyanin Analysis by High Performance Liquid Chromatography

The composition and quantification of the anthocyanins released from the lees was performed by injecting 20 µL of the filtered supernatant into a Waters 2695 liquid chromatograph (Waters, Milford, PA, USA). The chromatographic conditions used in this study were those described in Busse-Valverde et al. [42]. In the chromatographic analysis, a Licrochart RP-18 column (Merck, Darmstadt, Germany), 25 × 0.4 cm, particle size of 5 μm was used. The flow (0.8 mL/min) consisted of a gradient of two different phases: acetonitrile (100%) and a solution of formic acid at 4.5% (*v*/*v*) in high purity water. A diode array detector was used. Anthocyanins were quantified at 520 nm as malvidin-3-glucoside, using malvidin-3-glucoside chloride (Extrasynthese, Genay, France) as an external standard.

### 2.6. Soluble Polysaccharide Analysis by Size Exclusion Chromatography (SEC)

The mass distribution of the polysaccharides extracted from lees with the different treatments was analyzed by SEC. Two milliliters of the filtered supernatant were concentrated in a Centrivap concentrator (Labconco, Kansas City, MO, USA), and then dissolved in 250 µL of high purity water. It was necessary to dilute the sample due to the high amount of polysaccharides released, therefore 20 µL of the diluted sample was diluted again with 80 µL of high purity water. The volume of injection was 20 µL, the flow of 1 mL/min of LiNO3 (0.1 M), which passed through two columns connected in series (Shodex Ohpak KB-803 and KB-805; 0.8 × 30 cm, Showa Denkko, Tokyo, Japan). A refractometer detector was used (Waters 2414). The molar mass was determined using a calibration curve made with a special calibration kit (P-400, PM = 380,000; P-200, PM = 186,000; P-100, PM = 100,000; P-50, PM = 48,000; P-20, PM = 23,700; P-10, PM = 12,200; P-5, PM = 5800; Showa Denko K.K., Tokyo, Japan).

### 2.7. Statistical Analysis

The statistical analysis of the results was made with the statistical package Statgraphics Centurion. An analysis of variance (ANOVA) was made to determine differences among samples using a Test LSD to separate the means with a confidence level of 95%.

## 3. Results and Discussion

### 3.1. Determination of the Anthocyanins Liberated from the Lees

Table 1 shows the concentration of the anthocyanins extracted from the lees by the different applied treatments, analyzed by liquid chromatography (HPLC).

The aging of red wines usually implies the loss of monomeric anthocyanins. This is because they can be easily degraded, transformed into colorless forms and/or they could have polymerized into more stable forms. It has been reported that aging on lees reduces the degradation of anthocyanins, since the polysaccharides and mannoproteins released during yeast autolysis exert a protective effect on monomeric anthocyanins, and the red-blue color for which they are responsible, therefore, lasts longer [22]. However, and although this protective effect occurs, they also may promote a loss of anthocyanins due to an adsorption and precipitation phenomena [43]. Mazauric and Salmon [1] observed that a rather important part, that the initial wine colored polyphenols, especially those with a dominant blue color component, were strongly adsorbed on yeast lees and the possible recovery of these bound anthocyanins, so that they can again become part of the wine phenolic composition. This is the objective of this work.

The use of GLUC increased the concentration of monomeric anthocyanins, both non-acylated and acylated anthocyanins in the medium, especially the acylated ones that almost doubled their concentration. Morata et al. [15] reported that the acyl derivatives of anthocyanins (acetyl and p-coumaryl compounds) were more adsorbed than no-acyl derivatives by yeast cell walls of different *Saccharomyces* strains and GLUC could help their liberation.

Since the lees are not completely formed by yeast components, but they are also composed of a certain amount of precipitated plant cell walls, capable of binding phenolic compounds [44,45], a set of pectolytic enzymes (PEC) was also tested to promote the desorption of the phenolic compounds bound to these plant cell walls. The treatment with PEC did not increase the concentration of non-acylated anthocyanins when compared with the amounts measured in the control sample but it did so when the concentration of acylated anthocyanins was measured. The combination of both enzymes (PEC + GLUC) liberated almost twice as many acylated anthocyanins than when the enzymes were used separately, that is, they had an additive effect. The use of the enzymes PEC + GLUC also promoted a higher release of the pyranoanthocyanin vitisin A from lees. Vitisins are very stable pigments formed by the condensation of anthocyanins and yeast metabolites released during fermentation (pyruvic acid and acetaldehyde). Morata et al. [7] reported that vitisins (A and B) were adsorbed by the cell walls of most yeasts, although to a lower extent than monomeric anthocyanins. The recovery of this compound may have important significance for wine color, since these anthocyanins are very resistant to discoloration by SO_2_ [46] and express more intense colors than other pigments at pH 4 [47].

During the US treatments, several exposure times were tested. When looking at the sum of monomeric anthocyanin, the results showed that the ultrasound treatment reduced their concentration, especially for the longer treatments. Del Fresno et al. [34] observed a decrease in the anthocyanin content when US was applied to wines aged on lees compared to control wines, and they justified this loss of pigments due to the increase in dissolved oxygen found in the wines during the US treatment period. Another hypothesis can be proposed and it is that the cause of the degradation of monomeric non-acylated anthocyanins, which are more unstable than the acylated ones, could be due to the formation of oxidative radicals due to the sonication of the model solution. In fact, the quantity of liberated acylated anthocyanins increased with the duration of sonication time, and, although there are no significant differences between the three times assayed, differences were significant when compared with the concentration measured in the control solution, the concentration of acylated anthocyanins showed an increasing trend with sonication time.

Similarly, to acylated anthocyanins, sonication of lees during 120 min significantly increased the concentration of vitisin A. Liu et al. [38], while working with wine samples, used US to shorten the duration of aging on lees, and they determined that since the beginning of the aging on lees period, ultrasounds stimulated the formation of vitisins, since these compounds were not detected until the second month of the experiment in the control samples. However, in our case, being a model solution, the presence of vitisin A has to be the result of a desorption from the lees and not “the novo” formation.

Regarding the sum of all the detected anthocyanins, none of the US treatments showed significant differences with the control, however, the results indicate that a 120′ sonication time exerted the same effects as stirring the lees during 24 h, indicating that the process can save time in the treatment of lees.

When the different treatments were combined with US, interesting results were obtained. If the application of US was combined with stirring (as in the control sample), especially if the US was applied after a stirring period, the extraction of total anthocyanins significantly increased. Furthermore, all the combinations of US with enzymes extracted more anthocyanins than that measured in the control sample and when the ultrasound was used alone (US120′). The treatments in which the enzymes were used before the application of ultrasound extracted significantly more anthocyanins than those in which the sample was sonicated and then the enzyme was added. The better desorption capacity of the combinations in which the enzymes were used before the sonication of the sample could be due to the degradation created by the enzymes that enhanced the effect of the US on the lees. Working with wine samples, Osete-Alcaraz et al. [28] combined, during the maceration period, an initial treatment with a pectolytic enzyme followed by sonication of the pomace; this combination managed to increase the extraction of both anthocyanins and tannins from the grape skins to the fermenting must, confirming this mechanism of action. However, it should be noted that none of these enzyme and US combinations resulted in a significantly higher extraction of anthocyanins than when only stirring and US were applied (Stir-US).

### 3.2. Tannin Analysis by High Performance Liquid Chromatography and Size Exclusion Chromatography

Table 2 shows the results of the different treatments on the desorption of tannins, analyzed by the phloroglucinolysis method.

Our results showed that both the use of PEC and GLUC significantly increased the liberation of tannins compared to the control solution, and, as reported with total anthocyanins, the combination PEC + GLUC extracted the highest concentration of tannins. It is a well-known fact that both the plant cell walls and the yeast cell walls, which are in high concentrations in wine lees, have a great capacity to absorb tannins, absorbing even more than 50% of the tannins put in contact with them in model solutions [25,26,48]. Therefore, the greater effect of the combination of both enzymatic treatments, which act on both types of cell walls, was an expected result.

On the other hand, and regarding the use of ultrasound at different treatment times, it was clearly observed that increasing the treatment time also increased the extraction of total tannins. When US was applied for 120′, the concentration of desorbed tannins was higher than when the enzymes were used individually and similar to that found when PEC and GLUC were used in combination, although the treatment time was only 120′ instead of the 24 h needed for the enzymatic assays.

When looking at the different combinations with US, interestingly, the US120′ treatment extracted more tannins than when sonication was combined with 24 h of agitation (1ºStir-2ºUS and 1ºUS-2ºStir). The combination of US with GLUC did not improve the results of US working alone. Contrary to that, the use of PEC followed by US and the combination of PEC and GLU plus sonication led to the maximum recovery of tannins, indicating that the most effective combinations were those in which the PEC enzyme was used. It has been shown that the components of plant cell walls that have the highest affinity with tannins are pectins [49]. Our results could indicate that tannins bound to these are more easily extracted when a specific pectolytic enzyme was used than those bound to yeast cell walls, or that the amount of tannins bound to the grape cell wall was much higher than those bound to yeasts cell walls.

Regarding the mean degree of polymerization, there was a slight increase in this value in the liberated tannins when PEC + GLUC were used at the same time. It was also clearly observed that increasing the treatment time of the ultrasound treatments also increased their mDP. In the combined treatments, PEC enzyme (alone or in combination with GLUC) and sonication managed to extract the most polymerized tannins. Mazauric and Salmon [48] determined that there was no preferential adsorption of low or high polymeric size tannins, since they barely detected variation in the mDP of the tannins that remained in the wine after putting them in contact with yeast lees for a week. However, it has been reported that the plant cell walls do preferentially bind tannins with a higher degree of polymerization [25,26]. Therefore, techniques that degrade these plant cell walls will release those higher molecular weight tannins, such as observed when US and the pectolytic enzyme (PEC) were used.

The % of galloylation decreases significantly with the application of ultrasound (30′, 60′ and 90′) and the enzymatic combination (PEC + GLUC). In addition, %Gal decreased (in most of the cases) with combinations of ultrasounds and enzymes with respect to the control. There is an inverse relationship between the amount of total tannins extracted and the % of galloylation of these tannins, when tannins were more efficiently extracted, their % of galloylation was the lowest. It is likely that the galloylated tannins form the strongest unions with the plant and yeast cell walls, and by increasing the extraction of total tannins, those non-galloylated tannin increased more than the galloylated units, therefore, decreasing the galloylation percentage of the liberated tannins. In this way, Mazauric and Salmon [48] observed that polar tannins were preferentially adsorbed on yeast lees independent of their polymeric size.

**Figure 1:**
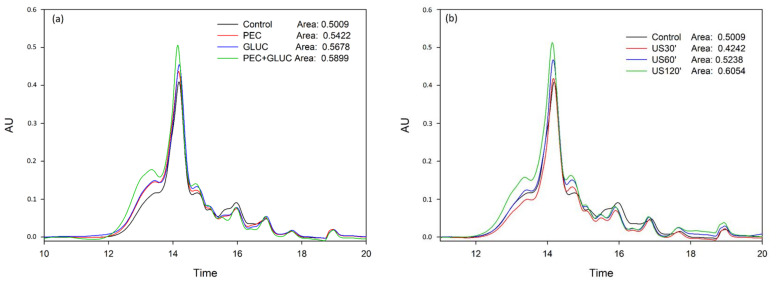
Mass distribution of the tannins released from lees analyzed by SEC in the elution time comprised between 12 and 20 min. (**a**) Comparison of tannins composition released with the enzyme treatments. (**b**) Comparison of tannins composition released with the US treatments. This figure was made in SigmaPlot 10.0.

**Figure 2:**
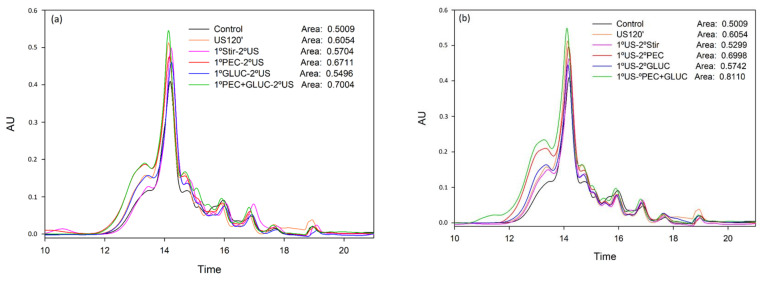
Mass distribution of the tannins released from lees analyzed by SEC in the elution time comprised between 12 and 20 min. (**a**) Comparison of tannins composition released when the enzyme treatments were added first and US treatment second. (**b**) Comparison of tannins composition released when the US treatment was used first and enzyme treatments were added second. This figure was made in SigmaPlot 10.0.

The analysis of tannins by phloroglucinolysis only provides information regarding easily depolymerizable tannins, mainly those non-oxidized tannins. To get a more complete image of the effect of the treatments on the molecular weight distribution of the desorbed compounds, the samples were analyzed by SEC. Figure 1 and Figure 2 show that the results obtained with this chromatographic analysis were comparable to those obtained in phloroglucinolysis analysis, since the same differences between samples were obtained and hardly any change was observed in the distribution of the molecular weights of the tannins due to the application of the treatments. The most important finding was that a clear increase in high molecular weight tannins was observed in the samples treated with the combination of PEC + GLUC, with or without sonication. This increase was also observed in the values of the mDP (Table 2), although it is more clearly seen in the SEC analysis, indicating that the use of both enzymes facilitated the liberation of high molecular weight tannins, more tightly bound to the cell walls, and even more when US was also applied.

### 3.3. Analysis of Soluble Polysaccharides by Size Exclusion Chromatography

The quantities of polysaccharides that were released to the medium due to the different treatments were also analyzed by size exclusion chromatography. This analysis was carried out to determine, beside the desorption of phenolic compounds, the capacity of the treatments, applied to the lees (composed of both plant cell walls and yeast cell walls), to liberate polysaccharides, which in fact, is the main objective of the aging on lees process.

**Figure 3:**
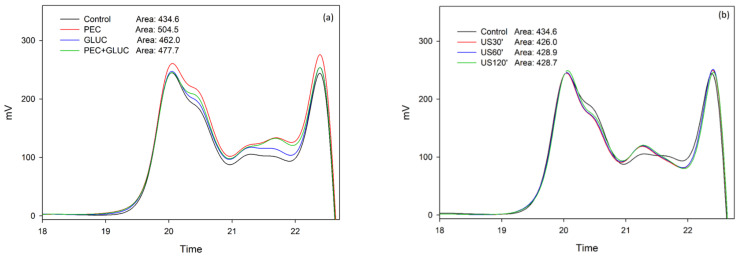
Mass distribution of the polysaccharides released from lees analyzed by SEC in the elution time comprised between 12 and 20 min. (**a**) Comparison of polysaccharides composition released with the enzyme treatments. (**b**) Comparison of polysaccharides composition released with the US treatments. This figure was made in SigmaPlot 10.0.

**Figure 4:**
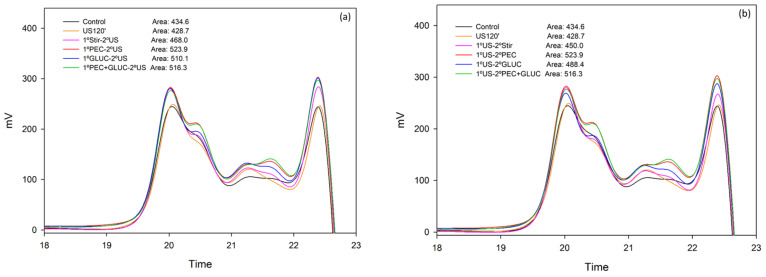
Mass distribution of the polysaccharides released from lees analyzed by SEC in the elution time comprised between 12 and 20 min. (**a**) Comparison of polysaccharides composition released when the enzyme treatments were added first and US treatment second. (**b**) Comparison of polysaccharides composition released when the US treatment was used first and enzyme treatments were added second. This figure was made in SigmaPlot 10.

The results shown in Figure 3 and Figure 4 indicate that the polysaccharides that are released from the lees in all the treatments, even in the control samples, had very low molecular weights. De Issepi et al. [37] studied the effect of ultrasound and enzymatic treatments (β-glucanase) in obtaining mannoproteins from lees, and they reported that although the extraction methods they adopted were designed to extract mannoproteins with molecular weights in the range of 5–800 kDa, when using these treatments, they obtained a large proportion of oligosaccharides in the extracts.

When only enzyme treatments were used, the pectolytic enzyme released the highest amount of polysaccharides. The capacity of this enzymatic preparation (composed of pectin lyase and polygalacturonase) to degrade plant cell walls and release soluble polysaccharides has been extensively studied in other studies [25,26,50]. In the study of Osete-Alcaraz et al. [25], cell walls from Monastrell grape skin were put in contact with different hydrolytic enzymes and it could be observed how the pectin-lyase extracted a very high quantity of soluble polysaccharides (especially low molecular weight polysaccharides) from the purified grape skin cell walls. Since the enzyme preparation used in this study contains large amount of this enzyme, we were not surprised by the high effectiveness of this enzymatic preparation in extracting low molecular weight polysaccharides.

Interestingly, when the two enzymes were used together (PEC + GLUC), a less amount of polysaccharides were released than when PEC acted individually. The same phenomenon occurred in the study by Osete-Alcaraz et al. [25], where they reported that the use of the enzyme pectin-lyase alone released twice the amount of soluble polysaccharides than when it was used in combination with other enzymes such as cellulase, xylanase or pectinmethylesterase. It is possible that a competition of the enzymes for the substrate could occur, partially inhibiting their action. The better action of PEC releasing polysaccharides could also indicate that there is a relatively large number of plant cell walls in the lees or that these cell walls more easily liberate polysaccharides, when compared with yeast cell walls.

The glucanase enzyme only released slightly higher quantities of polysaccharides than the stirred control solution. Palomero et al. [22] analyzed the composition of the polysaccharides of red wines to which β-Glucanase had been added. In their study, the addition of β-glucanase led to a degradation of the yeast cell walls, almost complete at 3 weeks whereas the controls needed up to 5 months, in addition, the polysaccharide profile was not the same as that obtained with conventional aging on lees since the fragments produced were generally smaller and of more uniform size. Probably, the short time the lees were in contact with the enzyme in this study (24 h), could account for the lack of effectiveness of the GLUC enzyme.

The US treatments released almost the same amount of soluble polysaccharides as the control, but in shorter times compared to the control sample (30, 60 or 120 min’ vs. 24 h in the case of the control samples). When stirring and ultrasound were combined (1ºStir-2ºUS and 1ºUS-2ºStir), the extraction of soluble polysaccharides was favored, in addition, barely any differences were observed related to the order in which each treatment was applied. Cacciola et al. [32] determined that the most important parameter in the ultrasound treatment for the extraction of soluble colloids is the treatment time, they obtained a linear growth in the extraction of soluble colloids as the treatment times increased. It is likely that if we had applied a longer treatment time in this study, the extraction of soluble polysaccharides presented by the control (shaking for 24 h) would have even been exceeded.

All the treatments where US and enzymes were combined released more polysaccharides than the control, the sonication of samples during 120 min and the enzymatic treatments applied individually. This result indicates that the combination of US and enzymes presented an additive effect for the extraction of polysaccharides from lees, as was found for the extraction of anthocyanin and tannin.

The order in which the US and enzymes were applied did not lead to significant differences, which coincided with the results obtained when studying the liberation of tannins. The treatments that led to the highest polysaccharide extraction were those in which the PEC enzyme was combined with US, with hardly any differences when used alone (1ºPEC-2ºUS and 1ºUS-2ºPEC) or together with glucanase (1ºPEC + GLUC-2ºUS and 1ºUS-2ºPEC + GLUC). This result was similar to that observed when the enzymes were used individually (without combining with the US) (Figure 3a), PEC + GLUC did not show greater extraction of soluble polysaccharides than PEC alone.

## 4. Conclusions

Aging on lees may not only be a process for increasing the liberation of polysaccharides for improving wine stability but also for increasing (or recovering) the wine phenolic content, by the desorption of those phenolics bound to plant and yeast cell walls. We have demonstrated that this liberation occurs and it can be boosted by techniques such as US and the addition of enzymes, which are already used for shortening the aging on the lees process.

The combination of the pectolytic enzymes and β-Glucanase was the most effective treatment in the liberation of anthocyanins, whereas the use of the pectolytic enzyme largely favored the liberation of tannins and also low molecular weight polysaccharides.

The application of US led, in only 120 min, to the same desorption of phenolic compounds than 24 h of sample stirring and the combination of US (120 min of treatment), GLUC and PEC showed, in general, an additive effect, increasing the extraction of phenolic compounds (tannins and anthocyanins) compared to individual treatments and also releasing a large quantity of low molecular weight polysaccharides, compounds of enological importance.

The results of this study, although it is a preliminary experience carried out on a model wine solution, indicated that it is possible that aging on lees could also improve the phenolic composition of a red wine, if techniques such as ultrasound or the addition of enzymes are applied during the process. Although the application of β-glucanase is commonly used during this process, enologists should also consider the application of a pectolytic enzyme to improve the whole process, liberation of polysaccharides and phenolic compounds.

## Figures and Tables

**Table 1:** Effect of the addition of enzymes and application of ultrasound in the release of anthocyanins from red wine lees. Characterization and quantification of anthocyanins in solution (µg/g ± standard deviation).

	Del (µg/g)	Cian (µg/g)	Pet (µg/g)	Peond (µg/g)	Malv (µg/g)	∑Mono (µg/g)	VitA (µg/g)	∑Acyl (µg/g)	∑Ant (µg/g)	∑Ant (mg/L)
Enzyme treatments										
Control	40.2 ± 1.9 a ^1^	10.4 ± 0.2 a	91.4 ± 2.4 a	49.8 ± 1.4 a	665.4 ± 9.8 a	857.3 ± 11.2 a	9.4 ± 0.2 a	36.9 ± 5.1 a	903.7 ± 15.8 a	56.5 ± 1.0 a
PEC	42.7 ± 1.3 a	10.8 ± 0.3 ab	97.1 ± 2.3 a	53.0 ± 1.0 b	676.3 ± 5.7 ab	879.9 ± 9.9 a	6.5 ± 0.4 a	60.4 ± 3.5 b	949.9 ± 13.8 b	59.4 ± 0.9 b
GLUC	55.0 ± 7.9 b	11.4 ± 0.2 b	108.9 ± 7.1 b	54.0 ± 0.5 b	692.9 ± 7.7 c	922.1 ± 16.0 b	10.6 ± 0.8 ab	61.0 ± 6.3 b	993.8 ± 17.3 c	62.1 ± 1.1 c
PEC+GLUC	59.3 ± 9.6 b	11.3 ± 0.5 b	111.4 ± 7.1 b	52.9 ± 1.5 b	682.4 ± 9.1 bc	917.1 ± 25.0 b	11.2 ± 0.9 b	102.1 ± 7.3 c	1030.7 ± 32.9 c	64.4 ± 2.1 c
Ultrasound treatments										
Control	40.2 ± 1.9 a	10.4 ± 0.3 a	91.4 ± 2.4 a	49.8 ± 1.4 a	665.4 ± 9.8 b	857.3 ± 11.2 b	9.4 ± 0.2 a	36.9 ± 5.1 a	903.7 ± 15.8 a	56.5 ± 1.0 a
US30′	61.9 ± 5.1 b	11.1 ± 0.7 a	104.4 ± 5.7 b	49.1 ± 3.1 a	573.4 ± 31.4 a	799.9 ± 45.9 ab	10.3 ± 0.6 ab	65.5 ± 17.1 b	875.7 ± 62.4 a	54.7 ± 3.9 a
US60′	60.6 ± 1.4 b	11.2 ± 0.7 a	102.9 ± 4.5 b	48.7 ± 2.5 a	571.0 ± 31.1 a	794.3 ± 39.7 a	10.1 ± 0.5 ab	68.3 ± 13.2 b	872.6 ± 29.8 a	54.5 ± 1.9 a
US120′	60.1 ± 1.9 b	11.0 ± 0.7 a	101.3 ± 3.2 b	47.8 ± 0.9 a	551.6 ± 9.5 a	771.7 ± 16.2 a	10.4 ± 0.4 b	84.8 ± 5.3 b	866.9 ± 21.9 a	54.2 ± 1.4 a
Combined treatments										
Control	40.2 ± 1.9 a	10.4 ± 0.3 a	91.4 ± 2.4 a	49.8 ± 1.4 a	665.4 ± 9.8 b	857.3 ± 11.2 b	9.4 ± 0.2 a	36.9 ± 5.1 a	903.7 ± 15.8 a	56.5 ± 1.0 a
US120′	60.1 ± 1.9 cd	11.0 ± 0.7 a	101.3 ± 3.2 b	47.8 ± 0.9 a	551.6 ± 9.5 a	771.7 ± 16.2 a	10.4 ± 0.4 b	84.8 ± 5.3 c	866.9 ± 21.9 a	54.2 ± 1.4 a
1ºStir-2ºUS	66.1 ± 3.1 de	13.3 ± 0.2 c	124.2 ± 1.3 cd	57.8 ± 0.5 b	747.0 ± 6.4 cde	1008.3 ± 2.3 d	12.3 ± 0.6 d	56.3 ± 0.3 b	1076.9 ± 1.5 bc	68.7 ± 2.5 cd
1ºUS-2ºStir	47.3 ± 1.4 b	11.9 ± 0.3 b	106.1 ± 1.1 b	57.4 ± 1.1 b	731.3 ± 8.6 cde	954.0 ± 11.4 c	10.4 ± 0.2 b	70.2 ± 8.6 bc	1034.7 ± 15.0 b	64.7 ± 0.9 b
1ºPEC-2ºUS	67.0 ± 6.8 e	14.2 ± 0.4 d	125.9 ± 3.4 b	49.3 ± 2.2 bc	754.1 ± 8.6 de	1020.6 ± 23.8 de	11.9 ± 0.9 d	112.8 ± 13.5 d	1145.3 ± 28.8 d	71.6 ± 1.8 d
1ºUS-2ºPEC	47.2 ± 2.8 b	12.1 ± 0.7 b	105.7 ± 5.9 b	56.9 ± 3.2 b	711.9 ± 30.8 c	933.7 ± 49.4 c	10.4 ± 0.7 b	110.2 ± 4.8 d	1054.3 ± 53.0 b	65.9 ± 3.3 bc
1ºGLUC-2ºUS	59.1 ± 3.7 c	13.8 ± 0.5 cd	122.0 ± 5.1 cd	62.0 ± 1.9 c	802.0 ± 36.9 f	1059.0 ± 32.0 e	12.0 ± 0.9 d	71.0 ± 16.7 bc	1142.0 ± 48.4 d	71.4 ± 3.0 d
1ºUS-2ºGLUC	46.7 ± 2.7 b	11.8 ± 0.1 b	105.7 ± 2.9 b	56.9 ± 0.2 b	723.9 ± 22.8 cd	945.1 ± 7.9 c	10.6 ± 0.1 bc	77.9 ± 11.1c	1033.6 ± 18.6 b	64.6 ± 1.2 b
1ºPEC+GLUC-2ºUS	48.3 ± 5.2 c	12.5 ± 0.5 b	118.4 ± 5.7 c	59.6 ± 2.5 bc	761.8 ± 2.4 e	1010.6 ± 36.4 d	11.4 ± 0.2 cd	108.5 ± 5.7 d	1130.5 ± 41.1 cd	70.7 ± 2.6 d
1ºUS-2ºPEC+GLUC	47.6 ± 2.7 b	12.2 ± 0.1 b	107.1 ± 3.4 b	57.3 ± 1.2 b	719.7 ± 15.0 cd	943.7 ± 22.3 c	10.5 ± 0.3 b	109.9 ± 12.0 d	1064.1 ± 34.2 b	66.5 ± 2.1 bc

Abbreviations: Del, delphinidin-3-glucoside; Cian, cyanidin-3-glucoside; Pet, petunidin-3-glucoside; Peond, peonidin-3-glucoside; Malv, malvidin-3-glucoside; ∑Mono, total concentration of monoglucoside anthocyanins; VitA, Vitisin A; ∑Acyl, total concentration of acylated anthocyanins; ∑Anthocyanins, total concentration of anthocyanins. All the values in the table are expressed in µg/g of lees. ^1^ Different letters in the same column, and for each treatment, mean statistically significant differences (*p* ≤ 0.05) (*n* = 3).

**Table 2:** Effect of the addition of enzymes and application of ultrasound in the release of tannins Figure 1. Different letters in the same column mean statistically significant differences (*p* ≤ 0.05) (*n* = 3).

Samples	TT (mg/L)	mPD	%Gal
Enzyme treatments			
Control	48.7 ± 2.5 a ^1^	2.95 ± 0.12 a	18.0 ± 1.8 b
PEC	63.8 ± 3.0 b	3.04 ± 0.03 a	15.9 ± 0.8 b
GLUC	59.1 ± 4.8 b	2.99 ± 0.11 a	15.4 ± 2.2 b
PEC + GLUC	91.8 ± 9.0 c	3.23 ± 0.10 b	11.5 ± 0.5 a
Ultrasound treatments			
Control	48.7 ± 2.5 a	2.95 ± 0.12 a	18.0 ± b
US30′	66.8 ± 14.6 ab	3.00 ± 0.05 ab	11.1 ± a
US60′	79.3 ± 12.5 bc	3.05 ± 0.03 ab	11.5 ± ab
US120′	88.0 ± 1.2 c	3.11 ± 0.03 b	12.8 ± a
Combined treatments			
Control	48.7 ± 2.5 a	2.95 ± 0.12 a	18.0 ± 1.8 de
US120′	88.0 ± 1.2 c	3.11 ± 0.03 ab	12.8 ± 0.5 abc
1ºStir-2ºUS	65.0 ± 7.5 b	2.98 ± 0.09 a	16.1 ± 2.5 cd
1ºUS-2ºStir	64.8 ± 4.3 b	3.30 ± 0.18 cd	15.6 ± 2.1 bcd
1ºPEC-2ºUS	103.9 ± 13.7 d	3.36 ± 0.15 cde	11.9 ± 0.9 ab
1ºUS-2ºPEC	99.9 ± 5.3 cd	3.52 ± 0.13 ef	10.5 ± 0.5 a
1ºGLUC-2ºUS	73.8 ± 12.9 b	3.22 ± 0.12 bc	19.7 ± 4.8 e
1ºUS-2ºGLUC	72.1 ± 7.6 b	3.39 ± 0.07 cde	15.2 ± 1.9 bcd
1ºPEC+GLUC-2ºUS	104.9 ± 5.9 d	3.44 ± 0.06 de	15.0 ± 0.6 bcd
1ºUS-2ºPEC+GLUC	104.3 ± 11.4 d	3.66 ± 0.10 f	12.0 ± 0.8 ab

Abbreviations: TT: total tannins measured by phloroglucinolysis method, mDP: mean degree of polymerization, %Gal: percentage of galloylation. ^1^ Different letters in the same column, and for each treatment, mean statistically significant differences (*p* ≤ 0.05) (*n* = 3).
